# The Evaluation for Alterations of DOM Components from Upstream to Downstream Flow of Rivers in Toyama (Japan) Using Three-Dimensional Excitation-Emission Matrix Fluorescence Spectroscopy

**DOI:** 10.3390/ijerph8051655

**Published:** 2011-05-19

**Authors:** Kazuto Sazawa, Masaki Tachi, Takatoshi Wakimoto, Takanori Kawakami, Noriko Hata, Shigeru Taguchi, Hideki Kuramitz

**Affiliations:** Department of Environmental Biology and Chemistry, Graduate School of Science and Engineering for Research, University of Toyama, Gofuku 3190, Toyama 930-8555, Japan; E-Mails: d1071303@ems.u-toyama.ac.jp (K.S.); beanshouse_orange@yahoo.co.jp (M.T.); sailing_470_4246@yahoo.co.jp (T.W.); river@sci.u-toyama.ac.jp (T.K.); noriko@sci.u-toyama.ac.jp (N.H.); taguchi@sci.u-toyama.ac.jp (S.T.)

**Keywords:** dissolved organic matter, three-dimensional excitation-emission matrix, humic-like components, protein-like components, river water, land usage

## Abstract

The dissolved organic matter (DOM) is one of the important factors for controlling water quality. The behavior and constitutions of DOM is related to the risk of human health because it is able to directly or indirectly affect the behavior, speciation and toxicity of various environmental pollutants. However, it is not easy to know the contents of DOM components without using various complicated and time consuming analytical methods because DOM is a complex mixture and usually exists at low concentration. Here, we describe the fluorescence properties of DOM components in water samples collected from four rivers in Toyama, Japan by means of the three-dimensional excitation-emission matrix (3DEEM) fluorescence spectroscopy. In order to evaluate the alterations of DOM components in each of the river during the flow from upstream to downstream, the patterns of relative fluorescence intensity (RFI) at six peaks which are originated from fluorophores including humic-like and protein-like components were investigated. The changes in the patterns of RFI values at each of the peak and the concentration of dissolved organic carbon (DOC) for each river water sample were discussed in connection with the differences of land use managements and basic water quality parameters, such as pH, EC, turbidity, Fe^3+^, T-N, NO_3_-N, T-P, PO_4_-P, chlorophyll *a*, DOC and N/P ratio. The DOC concentrations in the water samples collected from these rivers were relatively low (0.63–1.16 mg/L). Two main peaks which have a strong RFI value expressed a positive correlation with the DOC concentration (r = 0.557, 0.535). However, the correlations between the RFI values for other four peaks and the DOC concentration were below 0.287. The alterations of DOM components during the flow of a river from upstream to downstream were investigated from the changes in the patterns of RFI values for six fluorescent peaks. It was clarified that the great increase of RFI values in peak A and peak T from river water located in urban area showed high concentration of PO_4_-P and Fe^3+^, and low N/P ratio due to the high biological activities. The values of fluorescence index (FIX) and biological index (BIX) were as high as 1.60 and 0.72, respectively.

## 1. Introduction

There are many human activities that emit a wide variety of chemicals into the environment. Some of these chemicals are widely present in water environment and pose a threat to human health. Dissolved organic matter (DOM) is a mixture of organic environmental pollutants and a broad spectrum of humic substances that plays a key factor in affecting the behavior, speciation, solubility and toxicity of various organic and inorganic environmental pollutants [1,2]. Therefore, the behavior and constituents of DOM is very important in assessing the water quality and its potential risk to human health.

DOM consists of a complex mixture of organic components with a wide variety of chemical structures and molecular weights, which is operationally defined as the fraction of organic matter that can pass through a membrane filter with 0.45 μm pore diameter [3]. The DOM in aquatic environment represents one of the largest active organic carbon reservoirs in the biosphere [4] and originates from three different sources as follows: (1) Natural sources which include the production of humic substances in soil environment by biochemical and chemical reactions during the decay and transformation of plant and microbial remains. (2) Anthropogenic sources consisting of industrial and agricultural human activities. (3) Autochthonous production in an aquatic environment. Generally, the major components of DOM are humic substances which are heterogeneous mixtures of polydispersed materials known as fulvic acids and humic acids. The contribution of humic substances in DOM is typically 40–80% in river water and 15–80% in lake water [5]. The non-humic substances include natural components such as amino acids, proteins, lipids, carbohydrates, *etc.* [3], and artificial components such as detergents, pesticides, herbicides, *etc*. [6]. The DOM has a key role in the biogeochemical cycling and mobility of trace elements which are one of the important factors in determining the biotic function of an aquatic ecosystem. Therefore, the quantitative and qualitative analysis for DOM which has a strong effect directly and indirectly to water quality is critically important for evaluating aquatic environment.

Three-dimensional excitation-emission matrix (3DEEM) fluorescence spectroscopy is the result of sequential and simultaneous determination of three fluorescence parameters, *i.e.*, excitation wavelength, emission wavelength and fluorescence intensity. The 3DEEM method simultaneously detects several fluorophores with different fluorescence properties and has the following advantages: simplicity, speed, versatility, sensitivity and the fact it requires only a small volume of sample (*ca.* 3–4 mL). Therefore, this technique has been used as a powerful tool for a simple and high sensitive monitoring of DOM, including dissolved humic substances, which show strong fluorescent peak at around 355/450 nm (excitation/emission) [7]. The 3DEEM method has been extensively applied in characterization and monitoring of fluorophores in DOM of sewage water [8–10], fresh water [5,6,8,11–15] and the oceanic environment [9,10,14,16–19]. However, a few studies have also reported using the 3DEEM method to evaluate the alterations of DOM components during the flow of river water from upstream to downstream [5,6].

In this study, we describe the fluorescence properties of DOM including humic-like and protein-like components in water samples which were collected from four rivers in Toyama, Japan by means of the 3DEEM method. The alterations of DOM from upstream to downstream in each river were evaluated by the patterns of relative fluorescence intensity (RFI) at six peaks obtained from 3DEEM spectra for each river water sample and they are discussed by associating with the differences of land usage managements and changes in the water quality parameters, such as nutrients, chlorophyll *a* and DOC concentration.

## 2. Experimental Section

### 2.1. Study Sites and Sampling

**Figure 1.**
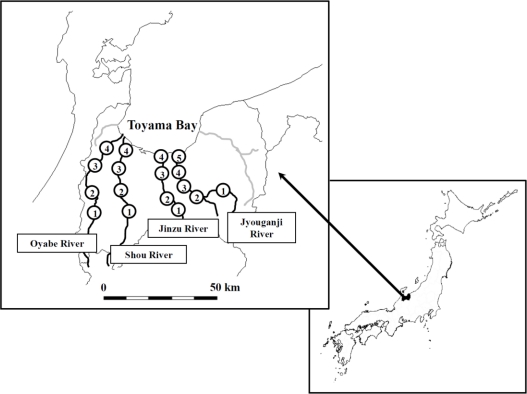
The location of Oyabe River, Shou River, Jinzu River, and Jyouganji River in Toyama, Japan and the sampling sites.

**Figure 2.**
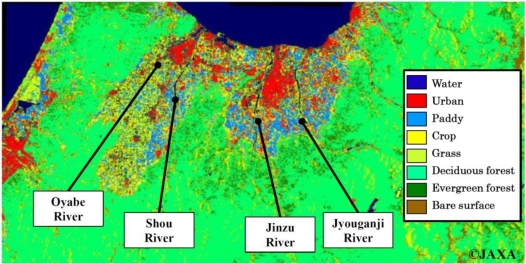
The land use management map for Toyama, Japan.

The four rivers, namely the Oyabe River, Shou River, Jinzu River and Jyouganji River, flow into Toyama Bay and they are specified as first-grade rivers by the Japanese Ministry of Land, Infrastructure, Transport and Tourism. The map of sampling sites and some information about land usage management in the area are shown in Figure 1 and Figure 2, respectively. The land usage management image measured by the Advanced Land Observing Satellite (ALOS) was obtained from the Japan Aerospace Exploration Agency (JAXA). The upstream of all four rivers are located in deciduous forest except for the Oyabe River (length is 68 km, basin area is 667 km^2^, flow rate is 58.3 m^3^/s) which is an anfractuous river which flows through an area of consisting of crops and paddies (site 1 to site 3), the downstream of this river passes through a sewage plant and a prime industrial area (site 3 to site 4). Moreover, two tributaries are located between site 1 and site 2 which are used for agriculture. Three tributaries between site 3 and site 4 are from the urban and industrial area. The population in the Oyabe River watershed is about 300,000. Shou River (length is 115 km, basin area is 1,189 km^2^, flow rate is 36.5 m^3^/s), which is located next to Oyabe River, flows through a paddy area and the watershed population is about 28,000. There is no significant tributary input to the Shou River. Jinzu River (length is 120 km, basin area of 2,720 km^2^, flow rate is 175.2 m^3^/s) has the largest amount of water flow and the greatest watershed population of 380,000. This river is well-known for “*Itai-itai disease*” which was caused by cadmium poisoning in the early twenty century. There are two tributaries flowing between site 2 and site 3, site 3 and site 4, respectively. A paddy area is located around site 2 and the urban area makes up site 3 and site 4. Jyouganji River (length is 56 km, basin area is 368 km^2^, flow rate 22.0 m^3^/s) is a torrential river which flows from an altitude of *ca.* 3,000 m. The watershed population of this river is about 30,000. The upstream of this river is located in a rich forest area of the Tateyama mountain range. As for the Jyouganji River, one tributary flows through a deciduous forest existing between site 1 and site 2.

The water samples were collected in 2 L polycarbonate bottles and stored on ice during transportation to the laboratory and immediately filtered through 0.45 μm pore diameter membrane filter (mixed cellulose ester, ADVANTEC) to measure the dissolved components.

### 2.2. Methodology

The river water samples were measured for pH, conductivity (EC), turbidity (FAU) and Fe^3+^ at the sampling location itself. The pH and EC value were measured using pH/conductivity meter (WM-22EP, TOA, Japan) and the turbidity and Fe^3+^ were measured by a HACH Meter (DR1850 colorimeter). The concentration of dissolved organic carbon (DOC) was measured by a high-temperature catalytic oxidation method. After the water sample was acidified (pH < 2) with HCl to remove dissolved inorganic carbon (DIC) by bubbling with pure air for 10 min, 50 μL of sample was injected into the TOC analyzer (TOC-5000A, Shimadzu, Japan). Total nitrogen (T-N) and dissolved nitrate ion (NO_3_-N) were determined by the HPLC system that included an LC-6A pump, SPD-6AV UV-VIS detector (220 nm, UV wave length, Shimadzu, Japan) and a TSK-gel DEAE-5PW column (7.5 mm inner diameter × 75 mm length, TOSOH, Japan) [20]. The mobile phase was 0.3 M NaCl and the flow rate was 1 mL min^−1^. The determination of T-N was carried out after degradation by potassium peroxodisulfate at 120 °C for 30 min. Total phosphorus (T-P) and dissolved orthophosphoric acid (PO_4_-P) were measured using spectrophotometric determination method according to Taguchi *et al*. [21]. Five hundred mL of the water sample was filtered with a glass fiber filter (0.45 μm pore diameter) to determine chlorophyll *a* (Chl. *a*). The fluorometric determination of Chl. *a* was performed with an extraction process using 10 mL of *N,N’*-dimethylformamide for 24 h at 4 °C as suggested by Porra *et al*. [22].

The 3DEEM spectrum was measured using a fluorescence spectrophotometer (Mode LS-55, Perkin Elmer). The scanning wave ranges were 200–600 nm for both of excitation and emission. Readings were collected at intervals of 10 nm excitation with 5 nm emission wavelengths using a scanning speed of 1,000 nm/min and photomultiplier voltage of 775 V. Pure water (Milli-Q, Millipore Co. Ltd) was used as the blank for all fluorescence analysis. Relative Fluorescence Intensity (RFI) was calibrated to be evaluated in quinine sulfate units (QSU); 1 QSU = 1 μg/L of quinine sulfate monohydrate in the solution of 0.05 M H_2_SO_4_ at excitation/emission (Ex./Em.) 355/450 nm. All the fluorescence spectra were applied for an inner-filter correction as according to McKnight *et al*. [12]. UV-Vis absorption spectrum is measured using a double beam spectrophotometer (U-2000A, HITACHI, Japan) equipped with a 1 cm quartz UV-visible cell.

## 3. Results and Discussion

### 3.1. Water Quality Parameters and DOC Concentration in the River Water

The water quality parameters, such as pH, EC, turbidity, Fe^3+^, T-N, NO_3_-N, T-P, PO_4_-P, Chl. *a*, DOC and ratio of T-P and T-N (N/P) for Oyabe River, Shou River, Jinzu River and Jyouganji River are shown in Table 1. The EC values of Oyabe River were found to increase gradually from upstream (0.607 μS/m, site 1) to downstream (1.098 μS/m, site 4). These changes of EC value are due to continuous discharge of ionic matter during the flow of river water. As for Jinzu River, the EC value increased from site 3 (0.752 μS/m) to site 4 (1.025 μS/m) and Jyouganji River showed an increment between site 1 (0.660 μS/m) and site 2 (0.832 μS/m). On the other hand, the EC values at Shou River hardly varied and were low compared with other rivers. Although Shou River is located close to Oyabe River, there was no increase of EC value as in the case of the Oyabe River. This might be a reflection of the differences in land usage management between both these rivers since there is no river flowing into Shou River which is used for agricultural and industrial activities, unlike in the Oyabe River. The change of EC value in Jyouganji River between site 1 and site 2 is probably due to the effect on the tributary inflow. This site is located in a paddy field area which may also contribute to the increase of the EC value. The EC value for Jinzu River increased significantly after flowing through an urban area between site 3 and site 4. In contrast with Jyouganji River, no increase of EC value was observed in Jinzu River between site 2 and site 3 when flowing through the paddy field. This could be due to the high amount of water in Jinzu River as compared to Jyouganji River. The turbidity values and Chl. *a* concentrations in these rivers did not show significant differences from upstream to downstream. This is probably due to the high flow rates of these rivers.

A high concentration of Fe^3+^ was detected downstream in all of these rivers and the values at Oyabe River and Shou River were the same and relatively higher than other ones. In Shou River, no increase of T-N, NO_3_-N, T-P and PO_4_-P concentrations from upstream to downstream was observed in contrast to the case of Oyabe River. However, the increase of these water parameters in Oyabe River was found at site 2 and site 4. If the high concentration of Fe^3+^ in the downstream of Oyabe River and Shou River depend on a geological characteristics around this land, the comparable high concentrations of T-N, NO_3_-N, T-P, PO_4_-P, Chl. *a* and DOC in Oyabe River are related with the differences of human activity and the land use managements between Oyabe River and Shou River. Moreover, the inflow to Oyabe River has a strong effect on its water quality.

The concentrations of T-N in Oyabe River and Jinzu River were relatively higher than that of the other two rivers and the highest value of T-N was found at site 4 in Jinzu River and second highest value was at site 4 in Oyabe River. The increase of NO_3_-N concentration was found at site 4 in Jinzu River and Oyabe River’s site 2 and site 4. In Oyabe River, the increase of NO_3_-N strongly contributes to the increase of T-N concentration at site 4. The maximum T-P concentrations in each river were as follows: 73.5 μg/L in Oyabe River, 40.3 μg/L in Shou River, 27.5 μg/L in Jinzu River and 11.5 μg/L in Jyouganji River. Both concentrations of T-P and PO_4_-P were relatively low in Jyouganji River and this could be associated to the low land use activity and watershed’s population at this catchment area as compared with other rivers. In Shou River, the T-P and PO_4_-P concentration decreased from upstream to downstream, in contrast to the Jinzu and Oyabe Rivers. In Jinzu River, the increase of T-P concentration was found only at site 4 located urban area, which also showed an increasing T-N concentration. The PO_4_-P concentration in Jinzu River hardly changed from upstream to downstream. Oyabe River has the highest concentration of T-P and PO_4_-P. The increase of these concentrations was found at the same site with the increase of NO_3_-N. Unlike in the case of Jinzu River, it can be said that the increase of PO_4_-P concentration contributes towards the increase of T-P concentration. According to the Annual Report on Environment published by Toyama Prefecture, the amount of nitrogen and phosphorus load discharged from Oyabe and Jinzu Rivers to Toyama bay is 55% and 50% of total amount, respectively [23]. Specifically, the sources for Jinzu River’s (annual average is 14 t/d) nitrogen load discharge are industrial (68%), domestic (4%) and non-point source (28%). On the other hand, 0.36 t/d of phosphorus load are discharged from Jinzu River and the breakdown in percentage for industrial, domestic and non-point source are 26%, 20% and 53% respectively. However, hardly any changes were observed in the concentrations of T-N, NO_3_-N, T-P and PO_4_-P between site 1 and site 3 of Jinzu River. Based on the map for land use managements shown in Figure 2, it seems that the high T-N, NO_3_^−^ and T-P concentrations at site 4 in Jinzu River is mainly due to the industrial activities located at this urban area. From Oyabe River, 10 t/d of nitrogen is discharged and of this 43%, 15%, and 47% originate from industrial, domestic and non-point sources, respectively. The phosphorus load discharge from Oyabe River is 0.84 t/d, which 2.3 times greater than that of Jinzu River. The breakdowns of phosphorus discharge in percentage for the sources are as follows: 43%, 23%, and 33% from industrial, domestic and non-point sources, respectively. As compared with Jinzu River, it is obvious that the effect of domestic source from the urban area is stronger in Oyabe River. This is a result of the sewage plant’s treatment which uses an activated sludge process to discharge high concentrations of orthophosphoric acid. Unlike the findings for Jinzu River, the increase of T-P values for this river was accompanied by the increase of PO_4_-P concentration at site 1 to site 2 and site 3 to site 4. From these results, it can be assumed that Oyabe River is probably strongly influenced by human activities, especially from domestic sources, taking place along this river as compared with the other rivers.

An increase of DOC concentration was only found at site 4 of Oyabe River and Jinzu River. Since the N/P ratio was low at site 4 in Oyabe River, unlike site 4 of Jinzu River, the increase of DOC concentration most probably related to the autochthonous (internal) production. The DOC concentrations of river water measured in this study were relatively low compared with the water samples used for the investigation by means of 3DEEM method as reported in previous studies (Maie *et al*., [15]: 8.28–11.2, Mostofa *et al.*, [5]: 0.52–3.30, [6]: 0.56–5.50 mg/L). Moreover, the changes of DOC concentration from upstream to downstream were also small compared with their previous reports. However, 3DEEM is known as a very sensitive semi-quantitative technique to evaluate even in marine environments where DOC concentration is less than 1 mg/L [8,17]. Therefore, it can be expected that the 3DEEM spectra are able to show the alternation of DOM components from upstream to downstream of rivers even at low concentration and also the small changes of DOC value.

### 3.2. Properties of Fluorescence Dissolved Organic Matter in the River Water

**Figure 3.**
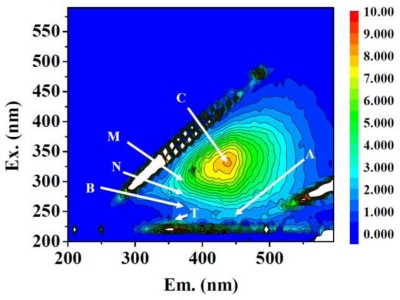
An example of the 3DEEM fluorescence spectrum for the river water sample collected from site 4 of Oyabe River. The bar chart next to the graph indicates contour intervals of the fluorescence intensity in the quinine sulfate normalization (QSU).

The optical properties of fluorescence dissolved organic matter in the river water samples were characterized from the point of intensity and position of fluorescence peaks obtained from 3DEEM spectrum. As an example, Figure 3 shows the 3DEEM spectrum and the peak positions (peak C, peak A, peak M, peak N, peak B and peak T) for the water sample obtained from Oyabe River’s site 4.

Coble *et al*. characterized the humic-like components by fluorescence properties as peak C (Ex./Em. = 320–360/420–460 nm), peak A (Ex./Em. = 260/380–460 nm) and peak M (Ex./Em. = 290–310/370–410 nm) in sea water [12]. Peak C and peak A which are represented as humic-like components exported from terrestrial sources are commonly detected simultaneously [17]. Peak A associates with the UVC-excited humic-like materials, which have the similar emission wavelength with the fluorophores given at peak C. The red-shift in the emission of peak C and peak A indicates that the fluorophores have rich aromaticity (extensive π-electron systems) and more functional groups [15,17]. Mostofa *et al*. have discussed that the photodegradation of fluorescence dissolved organic matter by solar radiation is responsible for the blue-shift of peak C [24]. It has been known that peak M is originated from marine humic substances [17] but recently it has also been reported that peak M is associated with microbe origin and the strong FI of peak M have been observed from sewage treatment water [8–10]. Peak N is related with phytoplankton production and it is not a humic-like peak [17]. Two fluorescent peaks correspond with amino acid in protein molecules. Peak B and peak T represents tyrosine-like and tryptophan-like fluorophores, respectively [14,16]. Peak T is also associated with biological production in surface waters [19,25]. Additional information on proteinaceous materials can be obtained from the peak position of peak T. This peak is known to shift depending on the molecular environments surrounding the tryptophan moiety. A blue-shift occurs when the tryptophan moiety is shielded from the surrounding water or hydrogen bounding groups [15]. However, polyphenol derivatives also have fluorescence properties in these regions [10].

Table 2 shows the relative fluorescence intensity (RFI) of each peak obtained from 3DEEM spectra of the water samples. The well defined peak C represented as humic-like components was observed as a major response in most of the 3DEEM spectra. Thus, the peak C positions for all the water samples are also shown in Table 2.

The RFI values of peak C observed in the water sample of Oyabe River were 1.4 to 4.4 times greater than the rest and were followed by the Jinzu River. The peak A as UVC-excited humic-like components was also detected in all river water samples, except at site 1 of Jinzu River and Jyouganji River. No drastic differences in the peak position of peak C were observed from the results obtained in this study. This indicates that the decomposition of UVA-exited humic-like components by photochemical reaction *etc*. hardly occurred in these rivers due to the fast flow rate of river. In the case of water samples collected from Oyabe River, the RFI values of peak M and peak N were significantly stronger than those from other river water samples and the peak T was clearly observed in the 3DEEM spectrum obtained downstream from site 4. From these RFI values obtained for each of the peaks, it can be said that the DOM components in Oyabe River are different from other rivers and it also seems that the DOM components in Oyabe River are more strongly influenced by biological actions as compared with other rivers.

**Figure 4.**
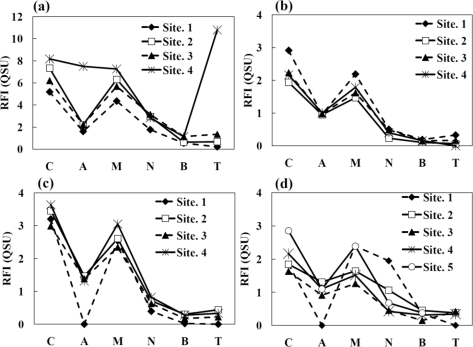
The patterns of RFI values at six fluorescent peaks (peak C, peak A, peak M, peak N, peak B and peak T) obtained from 3DEEM spectra for each water sample collected from (**a**) Oyabe River; (**b**) Shou River; (**c**) Jinzu River; and (**d**) Jyouganji River.

The evaluation for the alteration of DOM components during the flow was demonstrated by the patterns of RFI at six fluorescence peaks obtained from the 3DEEM spectra for each river water sample, as shown in Figure 4. The RFI values for peak C, peak A, peak M, peak N, peak B and peak T were read from the position at 320/420, 240/420, 290/370, 280/370, 270/370 and 220/345 (Ex./Em.) nm, respectively. The results from water samples of Oyabe River show that the RFI values of peak C gradually increased from upstream to downstream, and the value of peak A rapidly increased at site 4 [Figure 4(a)]. Moreover, the high RFI value at peak T which is related with tryptophan-like fluorophores had also risen at site 4. Although the DOC concentrations at site 1 and site 4 were similar, the patterns of RFI obtained from the six fluorescent peaks were drastically different. Namely, the qualitative changes of DOM components occurred between site 3 and site 4 where the urban area including industrial and sewage plant are located. As described above, there are two tributaries between site 1 and site 2 and three tributaries between site 3 and site 4 in Oyabe River. Although the concentration of NO_3_-N and PO_4_-P increased at both of site 2 and site 4, the qualitative changes of DOM components obtained from the patterns of RFI values were found at only urban area between site 3 and site 4 in Oyabe River. The RFI values of each of the peak obtained from 3DEEM spectrum for the finished water from the sewage plant are shown in Table 3. The main peak was peak C. The RFI values at all other peaks against the peak C were low compared with the patterns of RFI values at site 4 in Oyabe River [Figure 4 (a)]. Considering the low RFI values at peak T for the finished water, it can be assumed that the high RFI value at peak T of Oyabe River’s site 4 is most likely not directly related to the discharge from the sewage plant. It is unclear at this stage what causes the difference at the peak T’s RFI value (Oyabe River’s site 4). However, based on the information obtained from the land use management map and the fact that peak T is not observed on the mixture area of crops and paddies between site 1 and site 3 in Oyabe River, we could consider that to some extent, it is somehow related to industrial discharges. Taking into consideration the low value of N/P ratio and high concentration of PO_4_-P and Fe^3+^ at site 4 of Oyabe River, we could presume that the decomposition of DOM by biological actions in the river water might be linked to the generation of DOM components that resulted in the rise of peak T and peak A. In contrast to Oyabe River, changes in the patterns of RFI value at six fluorescent peaks from upstream to downstream were not observed in the results obtained from Shou River [Figure 4(b)]. Thus, it is expected that the quality of the DOM components in Shou River’s upstream is not so much different to the downstream. As shown in Table 1, the other water quality parameters of Shou River such as EC, T-N, NO_3_-N, Chl. *a* and DOC hardly changed. The change of DOM components in Jinzu River was observed between site 1 and site 2. The peak A which was not detected at site 1 appeared at site 2 [Figure 4(c)]. A similar phenomenon about peak A was also found at site 1 of Jyouganji River [Figure 4(d)]. This could be an influence from the paddy field which is located around these areas as according to the land use management map. As for the sample of paddy agricultural water, the respective RFI values for peak A, peak M and peak N were detected and are shown in Table 3. However, it is obvious that the RFI value was not high as expected for peak A. Therefore, the reason for the arisen of peak A cannot be clarified through this investigation. At site 4 in Jinzu River, an increment of DOC concentration was observed and this could a consequence of the industrial waste in the urban area. On the other hand, as in site 4 of Oyabe River, no qualitative changes of DOM components were observed from the patterns of RFI values. The difference observed at both site 4 of these two rivers is that the value of N/P ratio that acts as an indicator of biological activities was not low in Jinzu River, unlike in Oyabe River. This indicates that the generation of peak T and peak A observed in 3DEEM spectrum is suitable to detect the alteration of DOM components in water that has industrial discharge and low N/P ratio, *i.e.*, high biological activities.

### 3.3. Relationship of the Fluorescence Peak Intensity with DOC Concentration

**Figure 5.**
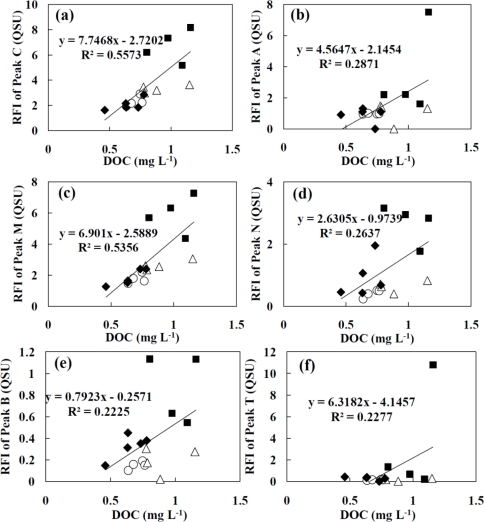
Relationship between fluorescence intensity of (**a**) peak C; (**b**) peak A; (**c**) peak M; (**d**) peak N; (**e**) peak B; and (**f**) peak T and concentration of DOC. The samples were collected from Oyabe River (▪), Shou River (○), Jinzu River (▵) and Jyouganji River (♦).

The relationships between the RFI value of six fluorescence peaks [(a) peak C; (b) peak A; (c) peak M; (d) peak N; (e) peak B and (f) peak T] and the concentration of DOC are shown in Figure 5.

In the previous report by Mostofa *et al*. [5], a high correlation between the RFI values of peak C, peak A and peak T obtained from river water and DOC concentration was found (peak C: r = 0.78, peak A: r = 0.81, and peak T: r = 0.78). In this study, the RFI values of peak C and peak M have a positive weak correlation with the concentration of DOC (r = 0.557 for peak C, r = 0.535 for peak M). However, the correlations between the RFI values for other peaks and the DOC concentration were low (r = 0.223–0.287). Although the DOC concentration of water samples in Oyabe River was similar with that obtained from Jinzu River, as shown in Table 1, the RFI values for peak C, peak M and peak N obtained from the sample water at site 2, site 3 and site 4 in Oyabe River were greater than the general trend of DOC concentrations. This indicates that the quality of DOM components that gave peak C, peak M and peak N is remarkably different in the downstream of Oyabe River than all other water samples collected from the sampling sites. The differences of DOM components could also be caused by the differences of the land use since a mixture of crop and paddy areas is located only in the upstream of Oyabe River. Furthermore, the RFI values for peak A and peak T at site 4 of Oyabe River showed great deviation that other water samples. The river water at this site showed high concentration of Fe^3+^, T-N, NO_3_-N, T-P, PO_4_-P and low N/P ratio. It is reported that the association of peak T with biological production in surface waters [19,25]. Therefore, if the risen peak A and peak T is not directly caused by the anthropogenic sources from industrial activities then the extremely high RFI values of peak A and peak T observed in the relation to DOM concentration most apparently related to the internal production including the decomposition of DOM components by high biological activity in the river water.

### 3.4. Fluorescence-Derived Indices

The fluorescence indices such as fluorescence index (FIX), humification index (HIX), and biological index (BIX) can be used to estimate the origin of DOM [26]. FIX is calculated from the ratio of the emission intensity at 450 nm to that at 500 nm, following excitation at 370 nm, provides a metric for distinguishing DOM derived from terrestrial and microbial sources. Terrestrial organic compounds, particularly lignin, are expected to contain more conjugated aromatic structures than microbially-derived substances. The FIX values of 1.4 or less indicate DOM of terrestrial origin and values of 1.9 or higher correspond to microbially-derived material [26,27], but the relative contribution of autochthonous and allochthonous substances cannot be discerned. BIX is the ratio of emission intensity at 380 nm and 430 nm at 310 nm of excitation can be used to assess the relative contribution of autochthonous DOM [28]. BIX values between 0.8 and 1.0 correspond to freshly produced DOM of biological or microbial origin, whereas values below *ca.* 0.6 are consider to contain little autochthonous DOM. HIX is an indicator of a material’s age and recalcitrance within a natural system [29]. The fresh DOM derived from plant biomass and animal manure shows low HIX values less than 5 and gradually increases with degree of humification. Highly humified organic substances are generally resistant to degradation and are expected to persist in the environment longer than low degree of humified substances. Water extractable DOM from soil and soil pore water has HIX values between 10 and 30 [30,31].

Table 4 shows the values of the respective values of FIX, HIX, and BIX for water samples collected from the above-mentioned rivers. The fluorescence indices values for three kind of water samples such as sewage treatment water, agricultural water collected from paddy area and Suwannee River fulvic acid are also described in the table. Based on the obtained FIX values, it can be assumed that most of the DOM components in the collected water samples consisted of terrestrially dominated substances. However, this observation was slightly different in the case of Oyabe River. Oyabe River showed relatively higher FIX values compared with the other rivers in this study. The FIX value at site 4 was the highest of all at 1.60. The high value of FIX (>1.4) such as the one obtained, indicates that DOM components at Oyabe River were mixed signatures of terrestrial and microbial sources. On the other hand, the contribution of autochthonous substances were higher in Jyouganji River and Oyabe River since the BIX values for both these rivers were higher than 0.6. The HIX values lower than 5 suggest that freshly produced DOM derived from plant biomass *etc*. were also obtained at downstream in Oyabe River. Therefore, this clarifies that the autochthonous microbially-derived substances contributed to the alteration of DOM components at site 4 in Oyabe River.

## 4. Conclusions

The results obtained in this study showed that the 3DEEM method is able to evaluate the alterations of DOM components during the flow of a river from upstream to downstream. The investigations were carried out at four rivers in Toyama, Japan. The changes in the patterns of RFI values at six different fluorescence peaks obtained from the 3DEEM spectra for each of the river water sample were investigated. Based on the information of land use managements and basic water quality parameters such as pH, EC, turbidity, Fe^3+^, T-N, NO_3_-N, T-P, PO_4_-P, Chl. *a*, DOC and N/P ratio, it is clarified that the RFI values of peak A and peak T greatly increased in industrial area with high concentration of Fe^3+^ and low N/P ratio, *i.e.*, high biological activities in river water. Namely, the surge in RFI values at peak A and peak T shows the alteration of DOM components by internal production. This study shows that 3DEEM is a useful tool to easily evaluate the alterations of DOM components in river water. However, the availability of information such as databases of 3DEEM spectra for various water sources and its relation with water quality parameters are not sufficient enough at the present stage. If more details regarding the contents of DOM components which produced the fluorescence response becomes available then the 3DEEM method will become a much more powerful tool to evaluate the water quality.

## Figures and Tables

**Table 1.** The parameters for water quality of Oyabe River, Shou River, Jinzu River and Jyouganji River.

**Samples**	**Site**	**pH**	**EC (μS/cm)**	**Turbidity (FAU)**	**Fe^3+^**	**T-N**	**NO_3_-N**	**T-P**	**PO_4_-P**	**Chl. *a* (μg/L)**	**DOC (mg/L)**	**N/P**

					**(mg/L)**			**(μg/L)**				
**Oyabe River**	1	8.01	0.607	1	0.11	0.86	0.25	13.6	7.12	0.16	1.09	63.4
	2	7.58	0.724	8	0.17	0.76	0.34	73.5	27.2	0.11	0.98	10.4
	3	7.68	0.887	8	0.19	0.83	0.33	36.4	18.9	0.12	0.80	22.8
	4	7.19	1.098	5	0.28	0.97	0.42	68.1	34.5	0.12	1.16	14.2

**Shou River**	1	7.37	0.541	6	0.14	0.51	0.17	40.3	9.93	0.07	0.74	12.6
	2	7.58	0.537	7	0.15	0.59	0.17	12.6	3.37	0.07	0.64	46.7
	3	7.76	0.547	N.D.	0.11	0.70	0.18	13.1	5.62	0.08	0.76	53.7
	4	7.74	0.555	N.D.	0.28	0.56	0.18	9.41	3.56	0.07	0.68	60.0

**Jinzu River**	1	7.26	0.741	N.D.	0.03	0.73	0.26	16.5	13.9	0.06	0.88	44.3
	2	7.33	0.758	N.D.	0.06	0.87	0.27	12.5	11.2	0.06	0.78	69.8
	3	7.33	0.752	N.D.	0.06	0.73	0.30	15.5	11.2	0.07	0.78	47.3
	4	7.22	1.025	N.D.	0.12	1.64	0.59	27.5	13.7	0.07	1.15	59.7

**Jyouganji River**	1	7.71	0.660	N.D.	0.09	0.59	0.24	8.54	2.62	0.06	0.73	69.1
	2	7.38	0.832	N.D.	0.10	0.57	0.20	8.42	3.56	0.05	0.63	67.8
	3	7.40	0.800	N.D.	0.07	0.57	0.24	8.57	2.43	0.05	0.46	66.5
	4	7.37	0.773	12	0.02	0.66	0.23	7.69	3.37	0.05	0.63	86.4
	5	7.33	0.827	4	0.17	0.79	0.24	11.5	5.81	0.03	0.78	68.7

**Table 2.** The RFI of each peak obtained from 3DEEM spectra of the river water samples. The RFI values for peak A, peak M, peak N, peak B and peak T were obtained from the peak’s position at 240/420, 290/370, 280/370, 270/370 and 220/345 (Ex./Em.) nm.

**Samples**	**Site**	**Peak C**		**Peak A**	**Peak M**	**Peak N**	**Peak B**	**Peak T**
		**Peak position (Ex/Em = nm)**	**RFI (QSU)**					
**Oyabe River**	1	330/440	5.19	1.61	4.35	1.77	0.55	0.21
	2	330/445	7.34	2.21	6.32	2.95	0.63	0.67
	3	330/440	6.21	2.20	5.69	3.15	1.13	1.35
	4	330/440	8.17	7.51	7.27	2.83	1.13	10.8

**Shou River**	1	330/445	2.91	0.96	2.19	0.51	0.19	0.33
	2	330/445	1.99	0.95	1.47	0.23	0.10	0.07
	3	330/445	2.24	0.98	1.62	0.49	0.15	0.17
	4	330/445	2.16	1.01	1.79	0.40	0.16	0.00

**Jinzu River**	1	340/445	3.21	0.00	2.55	0.39	0.02	0.00
	2	330/445	3.46	1.47	2.61	0.71	0.31	0.44
	3	330/440	2.98	1.38	2.35	0.63	0.17	0.22
	4	330/440	3.65	1.31	3.05	0.82	0.28	0.33

**Jyouganji River**	1	330/440	1.85	0.00	2.39	1.95	0.35	0.00
	2	340/410	2.40	1.31	1.64	1.06	0.45	0.38
	3	340/450	1.63	0.91	1.26	0.45	0.15	0.42
	4	330/445	2.17	1.10	2.40	0.43	0.31	0.34
	5	330/440	2.86	1.10	1.45	0.68	0.38	0.27

**Table 3.** The RFI of each peak obtained from 3DEEM spectra of the sewage treatment water and agricultural water for paddy. The RFI values for the peak A, peak M, peak N, peak B and peak T were selected from the peak position at 240/420, 290/370, 280/370, 270/370 and 220/345 (Ex./Em.) nm.

**Samples**	**Peak C**		**Peak A**	**Peak M**	**Peak N**	**Peak B**	**Peak T**
	**Peak position (Ex/Em = nm)**	**RFI (QSU)**					
**Sewage finished water**	340/440	66.0	5.56	28.5	21.3	13.3	0.65
**Agricultural water for paddies**	325/445	7.93	1.43	2.66	2.11	1.51	0.11

**Table 4.** The values of FIX, HIX, and BIX for Oyabe River, Shou River, Jinzu River, Jyouganji River, sewage treatment water, and agricultural water for paddy.

**Samples**	**Site**	**FIX**	**HIX**	**BIX**
**Oyabe R.**	1	1.39	6.23	0.55
	2	1.43	4.58	0.63
	3	1.47	3.03	0.69
	4	1.60	4.47	0.72

**Shou R.**	1	1.27	9.38	0.55
	2	1.28	28.92	0.55
	3	1.30	11.09	0.55
	4	1.29	13.52	0.54

**Jinzu R.**	1	1.46	356.66	0.56
	2	1.35	6.91	0.54
	3	1.34	10.82	0.57
	4	1.35	14.22	0.56

**Jyouganji R.**	1	1.28	4.21	0.96
	2	1.49	2.85	0.66
	3	1.25	5.58	0.53
	4	1.31	8.21	0.63
	5	1.39	6.31	0.57

**Sewage treatment water**		1.40	3.22	0.92
**Agricultural water for paddy**		0.92	5.32	0.61

**Suwannee River fulvic acid**		1.2	48	0.4
